# Gold Nanoparticles Augment N-Terminal Cleavage and Splicing Reactions in *Mycobacterium tuberculosis* SufB

**DOI:** 10.3389/fbioe.2021.773303

**Published:** 2021-12-23

**Authors:** Ananya Nanda, Sourya Subhra Nasker, Anoop K. Kushwaha, Deepak Kumar Ojha, Albert K. Dearden, Saroj K. Nayak, Sasmita Nayak

**Affiliations:** ^1^ School of Biotechnology, Kalinga Institute of Industrial Technology, Bhubaneswar, India; ^2^ School of Basic Sciences, Indian Institute of Technology Bhubaneswar, Bhubaneswar, India; ^3^ Departments of Physics and Astronomy, College of Arts and Sciences, University of South Carolina, Columbia, SC, United States

**Keywords:** gold nanoparticles, splicing regulation, splicing enhancement, NP–intein corona, SufB, *Mycobacterium tuberculosis*

## Abstract

Protein splicing is a self-catalyzed event where the intervening sequence intein cleaves off, joining the flanking exteins together to generate a functional protein. Attempts have been made to regulate the splicing rate through variations in temperature, pH, and metals. Although metal-regulated protein splicing has been more captivating to researchers, metals were shown to only inhibit splicing reactions that confine their application. This is the first study to show the effect of nanoparticles (NPs) on protein splicing. We found that gold nanoparticles (AuNPs) of various sizes can increase the splicing efficiency by more than 50% and the N-terminal cleavage efficiency by more than 45% in *Mycobacterium tuberculosis* SufB precursor protein. This study provides an effective strategy for engineering splicing-enhanced intein platforms. UV-vis absorption spectroscopy, isothermal titration calorimetry (ITC), and transmission electron microscopy (TEM) confirmed AuNP interaction with the native protein. Quantum mechanics/molecular mechanics (QM/MM) analysis suggested a significant reduction in the energy barrier at the N-terminal cleavage site in the presence of gold atom, strengthening our experimental evidence on heightened the N-terminal cleavage reaction. The encouraging observation of enhanced N-terminal cleavage and splicing reaction can have potential implementations from developing a rapid drug delivery system to designing a contemporary protein purification system.

## Introduction

**FIGURE 1:**
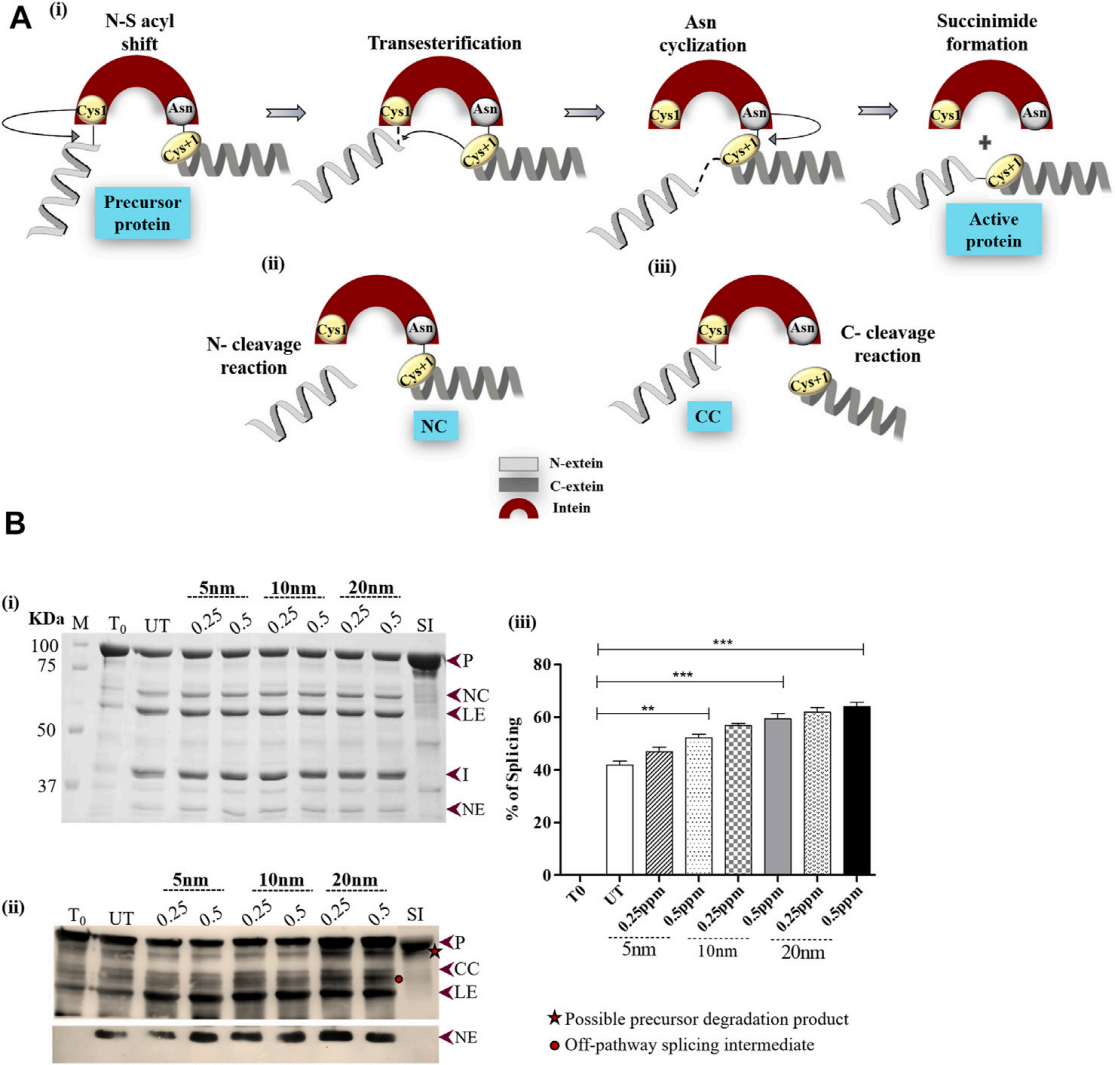
**(A)** Schematic diagram illustrating the reactions of intein splicing: i) Four sequential steps of intein splicing: nucleophilic attack by Cys1 and Cys+1 result in the formation of branched intermediate. Next, terminal Asn cyclization resolves the branched intermediate because of which intein splices out, ligating the flanking N- and C-exteins (active protein) (ii) and (iii) off-pathway splicing by-products; N- and C-terminal cleavage products (NC and CC), respectively. **(B)** Effect of AuNPs on *Mtu* SufB splicing. (i) Splicing products resulting from renaturation of *Mtu* SufB protein were resolved through 4–10% gradient SDS-PAGE. Lane 1 (T_0_): splicing products at time 0; Lane 2 (UT): splicing products in untreated SufB sample; Lanes 3–8: splicing products resulting from renaturation of *Mtu* SufB protein in presence of AuNPs (5, 10, and 20 nm) at different concentration (0.25 ppm, 0.5 ppm). Lane 9 (SI): splicing inactive SufB double mutant (C1A/N359A) is used as a negative control for splicing. ii) Western blot confirms the identity of protein products from Figure 1Bi. Anti-(His) antibodies detected the presence of 6x (His)-tagged P, CC, LE, and NE. NE is blotted separately with higher concentration of primary antibody. Two additional bands were identified: one below the precursor protein (possible precursor degradation product) and the other above ligated exteins (possible off-pathway splicing intermediate). iii**)** Bar graphs demonstrating statistically significant differences in splicing efficiency between untreated and NP-treated SufB [20 nm (*p* < 0.0001), 10 nm (*p* < 0.0001), and 5 nm (*p* = 0.004)]. All the experiments were performed in triplicates, and error bars represent (±1) SEM from 3 independent sets of experiments. The data shown are extracted from Figure 1Bi. P= precursor, CC = C-terminal cleavage product, NC = N-terminal cleavage product, LE = ligated extein, I = intein, and NE = N-extein.

Inteins are intervening polypeptides that auto-excise from host proteins, bonding the neighboring exteins in a process termed protein splicing (Perler et al., 1994; Paulus, 2000; Wu et al., 2014; Lennon et al., 2016; Lennon et al., 2018; Hoffmann et al., 2021). Protein splicing commences with a series of nucleophilic displacement reactions—an N/S or N/O acyl shift, a transesterification reaction, Asn cyclization, and an S/N or O/N acyl shift (Figure 1A) (Volkmann et al., 2013; Topilina et al., 2015; Nanda et al., 2020). Broadly, a splicing reaction generates splicing products (intein I, ligated exteins [LEs], or active protein) and/or off-pathway products [N-terminal (NC) and C-terminal (CC) cleavage products] (Figure 1A). Thus, intein insertion interrupts the functional domain of the precursor protein and abolishes the host protein activity, which is restored upon intein excision (Lennon et al., 2016; Sarmiento et al., 2019). Intein residues are numbered sequentially from 1 to n. N-extein residues are numbered as −1 to −n from the N-extein–intein junction such that the first residue of N-extein is numbered −1. Residues on C-extein are numbered +1 to + n from the C-intein–extein junction. Mutation of specific catalytic amino acid residues near splice junctions can convert inteins to isolated cleaving elements (Shi et al., 2013). A typical example constitutes the mutation of the first residue of intein (Cys1) to Ala1, which results in C-terminal cleavage, and the mutation of the last residue of intein (terminal Asn) to Ala, results in N-terminal cleavage (Paulus, 2000; Shemella et al., 2007; Shemella et al., 2011; Shah and Muir, 2014). The presence of intein in pathogenic organisms like *Staphylococcus aureus*, *Mycobacterium tuberculosis*, and *Plasmodium* sp. broadened the horizon of intein splicing regulation, opening avenues into the fabrication of intein-based pharmaceuticals (Perard and de Choudens, 2018).

To this day, intein splicing regulation has been broadly studied with transition metals such as zinc, copper, cadmium, cobalt, nickel, magnesium, calcium, and cisplatin. These studies reveal that metal coordination to intein critical residues takes control over the splicing mechanism by altering the conformation of the active site (Poland et al., 2000). Out of all the metal compounds, cisplatin regulates the splicing mechanism most potently, with an IC_50_ value of 1.67 µM (Zhang et al., 2011b; Chan et al., 2016b). Zinc reportedly has the strongest coordination to intein catalytic residues, with binding affinities measured at 3.4, 0.3, and 0.14 nM^−1^ in three *Mtu* RecA variants (Zhang et al., 2009). Zn exhibits 1 nM^−1^ binding affinity in a *Cne* Prp8 intein system (Green et al., 2019). The efficient inhibitory effect displayed by Cu^2+^ in *Mtu* RecA intein is a direct result of the strong coordination of Cu^2+^ to Cys1, with binding constant values varying from 0.098 to ∼0.16 nM. In the same system, Zn binding constants were in the range of 0.29–6.8 nM (Zhang et al., 2010b). Over the years, intein technology has shown promising advances in diversifying protein engineering methods, namely, the protein purification system (Chong and Perler, 2003; Sharma et al., 2006; Topilina and Mills, 2014; Guan and Chen, 2017). Despite addressing the shortcomings of conventional protein purification like omitting the affinity tag-removal process; intein-mediated purification platforms often run the risk of lower cleavage efficiency and uncontrollable cleavage that measure up to increased purification time with depreciating purification yield and efficacies (Xia et al., 2020).

Nanoparticles (NPs), especially due to their size (1–100 nm), promptly interact with biological systems to form protein “corona.” Nanoparticle protein corona (NP-PC) at the NP interface is a dynamic structure and is driven by forces like van der Waals, hydrophobic, and electrostatic interactions along with the binding affinity of protein, size, curvature, and surface charge of NPs (Casals et al., 2011; Zhu et al., 2012; Rahman et al., 2013; Sutkovic et al., 2016; García-Álvarez et al., 2018; Breznica et al., 2020; Park, 2020). Prior studies suggest that these factors not only affect the rate of protein adsorption at the nanoparticle interface but also modify the structure of the protein and function (Shemetov et al., 2012; Saptarshi et al., 2013; Wang et al., 2020). Thus, it is important to understand nanoparticle–protein interactions at the biomolecular level. In fact, spherical NP surfaces allow better flexibility and enhanced surface area to the adsorbed protein than planar surfaces (Verma and Stellacci, 2010). Although NPs can occasionally cause irreversible changes in the secondary structure of a protein, gold nanoparticles (AuNPs) were also shown to induce conformational changes in proteins without any perturbation to their overall structure (Worrall et al., 2006; Lacerda et al., 2010) (Wangoo et al., 2008; Busch et al., 2019). Several studies have reported the enhancement of protein’s biological activity when adsorbed onto nanoparticles (Vertegel et al., 2004; Deka et al., 2012; Saware et al., 2015; Peixto de Almeida et al., 2018; Tavanti et al., 2019). Kaur and Forrest further revealed that proteins adopt different orientations during adsorption that result in different levels of bioactivity. Moreover, they highlight that the size of nanospheres strongly influences the binding activity of the protein (Kaur and Forrest, 2012; Saptarshi et al., 2013). AuNPs are thus versatile and robust structures due to their excellent size tenability and physicochemical properties. The high surface energy of AuNPs significantly increases its reactivity compared to metals, which influences the interaction with proteins and nucleic acids (Gagner et al., 2011; Monopoli et al., 2011).

The use of metals in an intein-based system is limited due to its inhibitory effect. Nanoparticle interaction with proteins is an emerging yet intriguing area of research, given the higher reactivity of nanoparticles than bulk metals. This prompted us to investigate the effect of nanoparticles on *Mtu* SufB splicing and cleavage reactions, which broadens its application in protein engineering and protein purification technology.

We report the first study on the activity of AuNPs on splicing and N-terminal cleavage reactions of *Mycobacterium tuberculosis* (*Mtu*) SufB precursor protein. To check the versatility of NPs, we tested AuNPs of sizes 5, 10, and 20 nm (Wang et al., 2015; Tian et al., 2017; Contini et al., 2020). Our study demonstrates a significant increase in splicing and N-terminal cleavage efficiencies in the presence of AuNPs of various sizes. The splicing and cleavage products were visualized by SDS-PAGE and further confirmed by Western blot. Although convincing results were obtained for 20-nm NPs, followed by 10-nm NPs, AuNPs of size 5 nm exhibited the least effect. Protein–AuNP interaction was confirmed by UV-vis spectrophotometry. Isothermal titration calorimetry (ITC) analysis provided comparative data on the affinity of test protein (*Mtu* SufB) towards the AuNPs. Transmission electron microscopy (TEM) detected AuNP–protein interaction, leading to “corona” formation. Quantum mechanics/molecular mechanics (QM/MM) analysis under solvent conditions allowed for a mechanistic understanding of the AuNP effect on *Mtu* SufB protein cleavage and splicing reactions.

## Materials and Methods

### Gold Nanoparticles

AuNPs of different sizes were procured from Sigma Aldrich: 5 nm (Product number 756528), 10 nm (Product number 752584), and 20 nm (Product number 753610).

### Protein Overexpression and Purification

The plasmids used in this study are previously engineered, and the details are mentioned elsewhere (Panda et al., 2021). *Mtu* SufB wild type, splicing inactive double mutant (C1A/N359A) SufB (negative control), and SufB intein all carrying N-terminal 6X (His) tag were overexpressed in BL21 (DE3) *E. coli* cells via IPTG (sigma 367-93-1) induction. Cells were resuspended in lysis buffer (20 mM sodium phosphate, 0.5 M NaCl, pH 7.4) and lysed via a tip sonicator (Sonics vibra cell VCX-130). The overexpressed proteins were isolated from inclusion bodies (IBs) via centrifugation. The IB materials were solubilized in 8 M urea (Merck, 1084870500) buffer (lysis buffer, 8 M urea, 20 mM of imidazole (MP–biochemicals-288-32-4) and centrifuged at 16,500x g for 20 min to collect the supernatant (Friedel et al., 2019a). Solubilized test proteins were purified using a Ni-NTA affinity column (Ni-NTA His trap, HP GE healthcare life sciences- 17524802). Prior to sample application, columns were equilibrated with binding buffer (20 mM sodium phosphate, 0.5 M NaCl,40 mM imidazole) and after sample loading, the columns were washed several times (15 CV) in binding buffer. Finally, test proteins were eluted as purified fractions in elution buffer (20 mM sodium phosphate, 0.5 M NaCl, 500 mM imidazole), followed by quantification via Bradford’s assay.

### *In Vitro* Protein Refolding and Splicing Analysis

For splicing assay, 2.5 µM of denatured test protein was refolded in refolding buffer (20 mM sodium phosphate, 0.5 M NaCl, 0.5 M Arginine) and 2 mM TCEP-HCl (sigma- 51805-45-9) for 4 hours, in the presence and absence of AuNPs (0.25 and 0.5 ppm) at 25°C (Mills et al., 1998; Mills and Paulus, 2001; Gangopadhyay et al., 2003; Zhang et al., 2011a; Yamaguchi and Miyazaki, 2014; Chan et al., 2016a). The 0 h sample was obtained by collecting the sample prior to renaturation and abrupt addition of loading dye (0.1% bromophenol blue, 50% glycerol, β-mercaptoethanol, 10% SDS, and Tris 6.8), followed by rapid freezing at −20°C.

The N-terminal cleavage assay for the purified proteins was conducted following the aforementioned experimental steps. Denatured test proteins were allowed to refold in the presence of AuNPs at a concentration of 0.5 ppm for 1 h. Samples were taken out at different periods (10, 20, 30, and 60 mins) followed by abrupt addition of loading dye and boiling at 95°C for 5 mins. Products from splicing and cleavage reactions were resolved through 4–10% gradient SDS-PAGE. Protein bands were stained with Coomassie brilliant blue R-250, and densitometric analysis was performed using GelQuant.NET biochemical solutions software. The splicing and cleavage efficiencies were calculated as percentage of the ratio of total splicing product (LE and I) over total proteins (LE + I + P) and total N-terminal cleavage product (NE + NC) over total proteins (NE + NC + P), respectively. The 0 h splicing and cleavage value(s) were subtracted each time for baseline correction. The results were analyzed using one-way ANOVA and plotted using GraphPad Prism version 5.01 for Windows, GraphPad Software, San Diego, California, United States, www.graphpad.com.

### Western Blot

Western blot analysis was performed by using an anti-His antibody (Invitrogen, LOT 1902132) to confirm the identity of splicing and cleavage products. The test proteins were transferred from SDS gel to PVDF membrane (Millipore, IPVH 00010); at 50V for 2 h. Next, the blot was blocked with 5% skim milk for 2 h at 25°C, washed with 1X TBST, and incubated with primary anti-His antibody (abgenex-32–6116) at 1:5,000 dilutions for 16 h at 4°C. In the final step, the blot was washed with 1XTBST and incubated with secondary antibody (anti-mouse, abgenex 11-301) at 1:8,000 dilutions for 2.5 h at 25°C. The N-extein band was detected using a higher dilution of the primary (1: 2,500) and secondary antibodies (1:6000). The blot was developed using enhanced chemiluminescence substrate (ECL) (abcam, ab65628).

### UV–Visible Spectroscopy

Purified SufB intein was incubated with AuNPs (20, 10, and 5 nm) at a concentration of 0.5 ppm for 4 h. Untreated SufB intein was taken as a control against each treated sample. The samples were scanned via UV–visible spectroscopy (UV-1800 Shimadzu) at 25°C in the visible region of spectrum 200–800 nm. To eliminate the interference of buffer nanoparticle interaction, the refolding buffer (20 mM sodium phosphate, 0.5 M NaCl, 0.5 M arginine + 8 M urea) was incubated with the AuNPs under similar experimental conditions and then scanned against respective test samples. The sole buffer solution was considered as the blank and used for baseline correction. The data were analyzed by plotting an absorbance vs wavelength graph in Origin (Pro) 8.5, OriginLab Corporation, Northampton, MA, United States.

### Isothermal Titration Calorimetry

ITC measurement was carried out at 25°C on MicroCal ITC200, Malvern. SufB intein was overexpressed and purified under native conditions. The protein was buffer exchanged to 0.1 M PBS (pH 7.4) by dialysis as the ligands (AuNPs) were in the same buffer. The ligand concentration was maintained 10 times higher than that of the protein to get the proper saturation. In total, 2 μl of AuNP solution was injected into the sample cell and allowed to titrate for 4 s. Within every injection, a 120-s time interval was maintained for equilibration. Then AuNP sample was loaded in a syringe and titrated against SufB intein for 30 cycles. The heat released or absorbed during the reaction was measured by an instrument. The titration of AuNP to the buffer solution was carried out along with each experiment as a reference for baseline corrections.

The reaction parameters for nanoparticle–protein interactions were obtained by fitting the titration graph of each nanoparticle against the protein in Origin (Pro) 8.5, OriginLab Corporation, Northampton, MA, United States. A baseline correction was applied to each experiment by subtracting data from titration of the AuNP solution into the buffer blank correlating to the heat of dilution (Zhang et al., 2010a). The binding isotherm was fitted to the data using a linear fit. The detected change in enthalpy and binding constants were obtained from the best-fit parameters.

### TEM Analysis

Protein corona formed by protein–AuNP interaction was visualized by using a transmission electron microscope (Jeol JEM-1400). SufB intein was overexpressed and purified under native conditions. The AuNP was allowed to interact with the protein for 4 h. The protein–AuNP complex was harvested and washed twice with 0.1 M PBS to remove the unbound protein from the reaction mixture. Bare AuNPs in 0.1 M PBS solution were used as controls against protein adsorption on the AuNP surface. The protein–AuNP complex pellet was resuspended and fixed with 2.5% glutaraldehyde for 1 h. For TEM analysis, 5 μl of the AuNP complex was placed onto the carbon-coated copper grid and imaged under a TEM, with an acceleration voltage of 120 kV (Sasidharan et al., 2015).

### Computational Analysis

**FIGURE 6:**
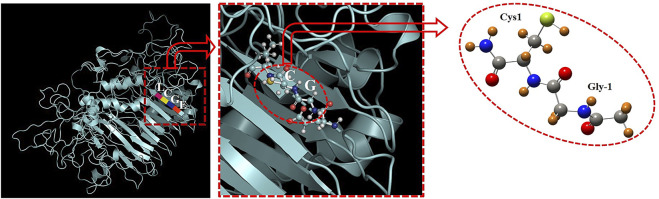
3D structure of *Mtu* full-length SufB precursor is obtained from Model Archive [(https://www.modelarchive.org/doi/10.5452/ma-x807d, Access code-6pmXRNkvwR] (Panda et al., 2021). EGCL represents the amino acid residues from 251 to 254. The structural arrangement of Gly252 (Gly-1) and Cys253 (Cys1) at the N-splice site are encircled in red.

The structure of *Mtu* SufB precursor has been depicted in Figure 6, where the amino acid sequence, 251–254, has been shown as EGCL (Panda et al., 2021). According to the previous model (Dearden et al., 2013; Dearden, 2014), we have selected the active site (Gly-Cys) with capping of the –CH3 (carboxylic terminal) and –CONH_2_ group (amide terminal), as shown in Figure 6. The selected active site (i.e., Gly-Cys) has been treated with the first principle–based density functional theory which is implemented on the Gaussian 09 computational code (Frisch et al., 2009). The Gly-Cys structure has been relaxed with full freedom of movement with the fixing of the dihedral angles at the B3LYP/6-311+G (d, p) level of theory. The dihedral angle constraints are necessary because of the removal of the surrounding protein system. During the geometry relaxations, average force and average changes over distance were fixed to the value 3 × 10^–4^ Hartree/Bohr and 1.2 × 10^–3^ Bohr, respectively. In the case of the induction of a Au atom, it was fixed at a distance of 2.3 Å away from the attacking sulfur (of Cys1), on the basis of the earlier experimental study, where zinc was found to be closer to the C-terminal catalytic cysteine (Sun et al., 2005). With the nearby Au atom, the active site system is relaxed with aforementioned constraints at the functional, B3LYP, and basis set LANL2DZ for Au, and 6-311+G(d, p) for other atoms. Furthermore, the optimized structure of the system in vacuum is treated with the polarized continuum model for solvent inclusion and calculation. In the case of PCM model, when a molecule is placed in the dielectric medium, spherical cavities are created around the atoms. These dielectric media are characterized by a dielectric constant (ε), for example, ε = 1, for vacuum as default and ε = 78.35 for water in solvent case. A previous study has found the dielectric constant of protein to be ∼10; therefore, in our calculation, we have used 10 as the dielectric constant for the subsequent investigations. For the reaction barrier calculations, the transition states were found using the synchronous transit and quasi-Newton method, which was then refined with the rational function optimization using the Berny algorithm. The presence of an imaginary frequency mode confirms the TS of both of the systems, that is, Au-induced and without Au system. For a detailed investigation of the reaction barrier, we perform the Mulliken charge analysis which is implemented in Gaussian 09.

## Results and Discussion

### Gold Nanoparticles Augment Splicing Reaction in *Mtu* SufB Protein

It has been widely accepted that AuNPs interact with proteins in a biological medium to form protein corona (PC). Factors like local environment; concentration; and affinity of protein, size, shape, and surface charge of AuNPs affect the kinetics of protein adsorption (Kaur and Forrest, 2012; Wang et al., 2015; Nicoară et al., 2019). We performed *in vitro* refolding of *Mtu* SufB (Materials and Methods Section) in the presence of AuNPs (0.25 and 0.5 ppm) of various sizes (5, 10, and 20 nm), to check the effect on the splicing reaction. Optimum AuNP concentrations were selected after performing gradient assay to evaluate the effect of differing concentrations of AuNPs on SufB splicing (Supplementary Figure S1).

After 4 h of refolding, the protein was resolved through 4–10% gradient SDS-PAGE. Splicing and cleavage reactions gave rise to precursor (P; 95.98 kDa), N-cleavage (NC; 66 kDa) product, N-extein (NE; 29.9 kDa), ligated extein (LE; 55.7 kDa), and intein (I; 40.2 kDa) (Figure 1Bi). None of these products were observed in splicing inactive *Mtu* SufB double mutant (SI, C1A/N359A), which was used as a negative control for protein splicing and to check the effect of the AuNPs on the stability of the precursor protein (Figures 1Bi,ii, 2C, 3C; Supplementary Figure S2). Previous studies have reported that alanine substitution in place of catalytic residues Cys1 and Terminal Asn completely abolishes splicing activity (Zhang et al., 2015). C-terminal cleavage (CC; 70.12 kDa) products and C-extein (CE; 25.7 kDa) were not detected in SDS-PAGE possibly due to protein degradation. SufB precursor protein (P), splicing, and cleavage products (CC, LE, and NE) carrying the N-terminal 6X (His) tag were further confirmed via Western blot using anti-His antibodies (Figure 1Bii). Two additional bands were detected, possibly precursor degradation product below the precursor protein and splicing intermediate product above LE (Figure 1Bii). The aforementioned bands were not visible with *Mtu* SufB intein treated under similar experimental conditions (Supplementary Figure S3B). The identity of splicing and cleavage products were confirmed by MALDI TOF/TOF Mass spectrometry and the results were submitted to the ProteomeXchange Consortium via the PRIDE with data ID PXD015199 (Submission details are provided in the Supplementary Material (Page S7).) (Panda et al., 2021).

**FIGURE 2:**
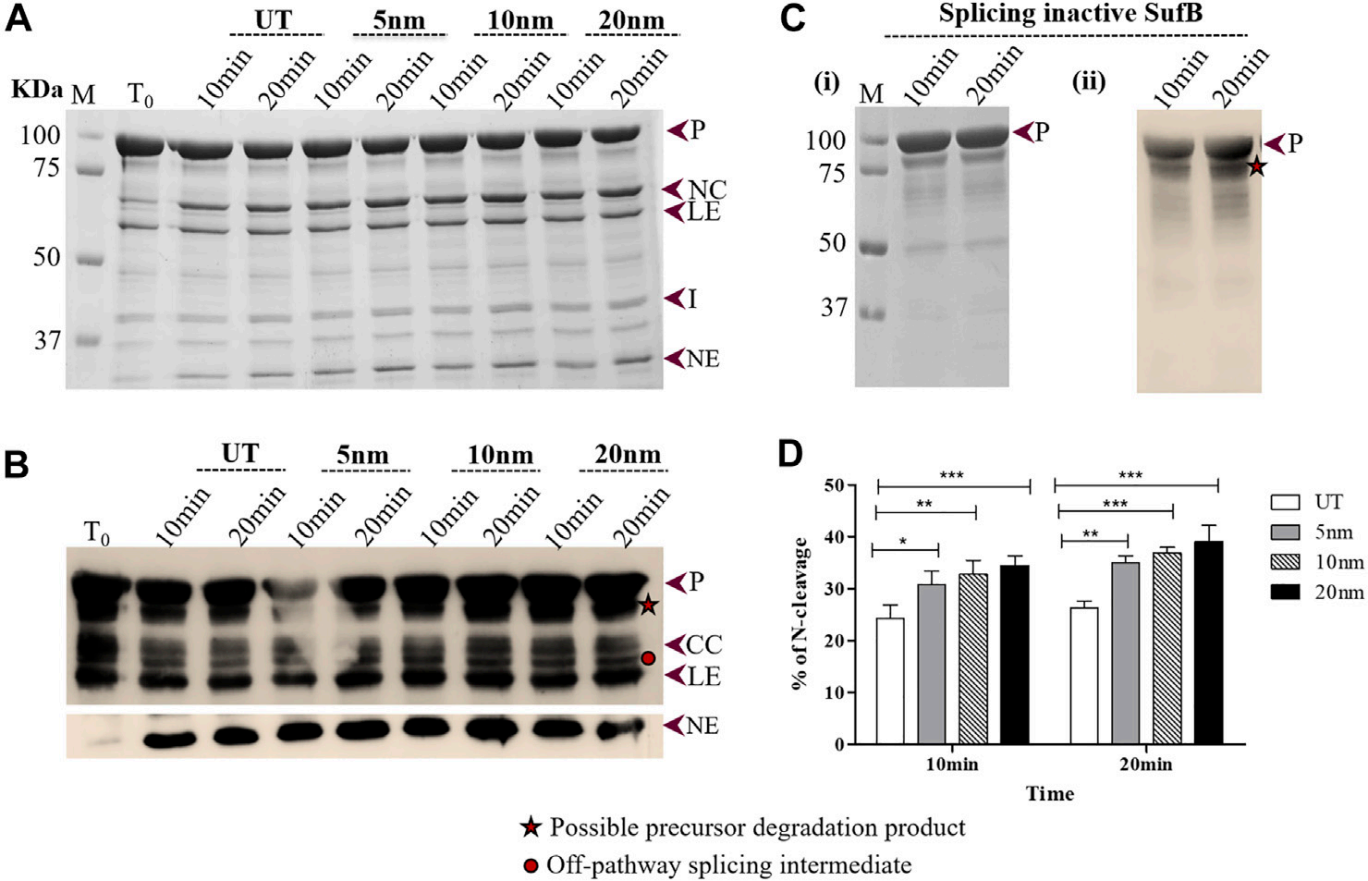
Effect of AuNPs on the N-terminal cleavage reaction until 20 mints following SufB renaturation: **(A)** N-terminal cleavage products resulting from refolding of *Mtu* SufB protein over different periods (10 and 20 minutes) were resolved through 4–10% gradient SDS-PAGE. Lane 1 (T_0_): cleavage products at time 0; Lanes 2,3 (UT): cleavage products in untreated SufB sample over specified time; Lanes 4–9: N-terminal cleavage products in presence of 0.5 ppm of AuNPs (5, 10, and 20 nm) after 10 and 20 minutes of refolding reaction. **(B)** Western blot confirms the identity of protein products from Figure 2A. Anti-(His) antibodies detected the presence of 6x (His)-tagged P, CC, LE, and NE. NE is blotted separately with higher concentration of primary antibody. Two additional bands were identified: one below the precursor protein (possible precursor degradation product) and the other above ligated exteins (possible off-pathway splicing intermediate). **(C)** (i) and (ii) SDS gel and western blot (anti-His antibody) images of splicing inactive SufB double mutant (C1A/N359A) that is used as a negative control. **(D)** Bar graphs demonstrating statistically significant differences in N-terminal cleavage efficiency between untreated and NPs treated SufB. All the experiments were performed in triplicates and error bars represent (±1) SEM from 3 independent sets of experiments. The data shown are extracted from Figure 2A. P= precursor, CC = C-terminal cleavage product, NC = N-terminal cleavage product, LE = ligated extein, I = intein, and NE = N-extein.

**FIGURE 3:**
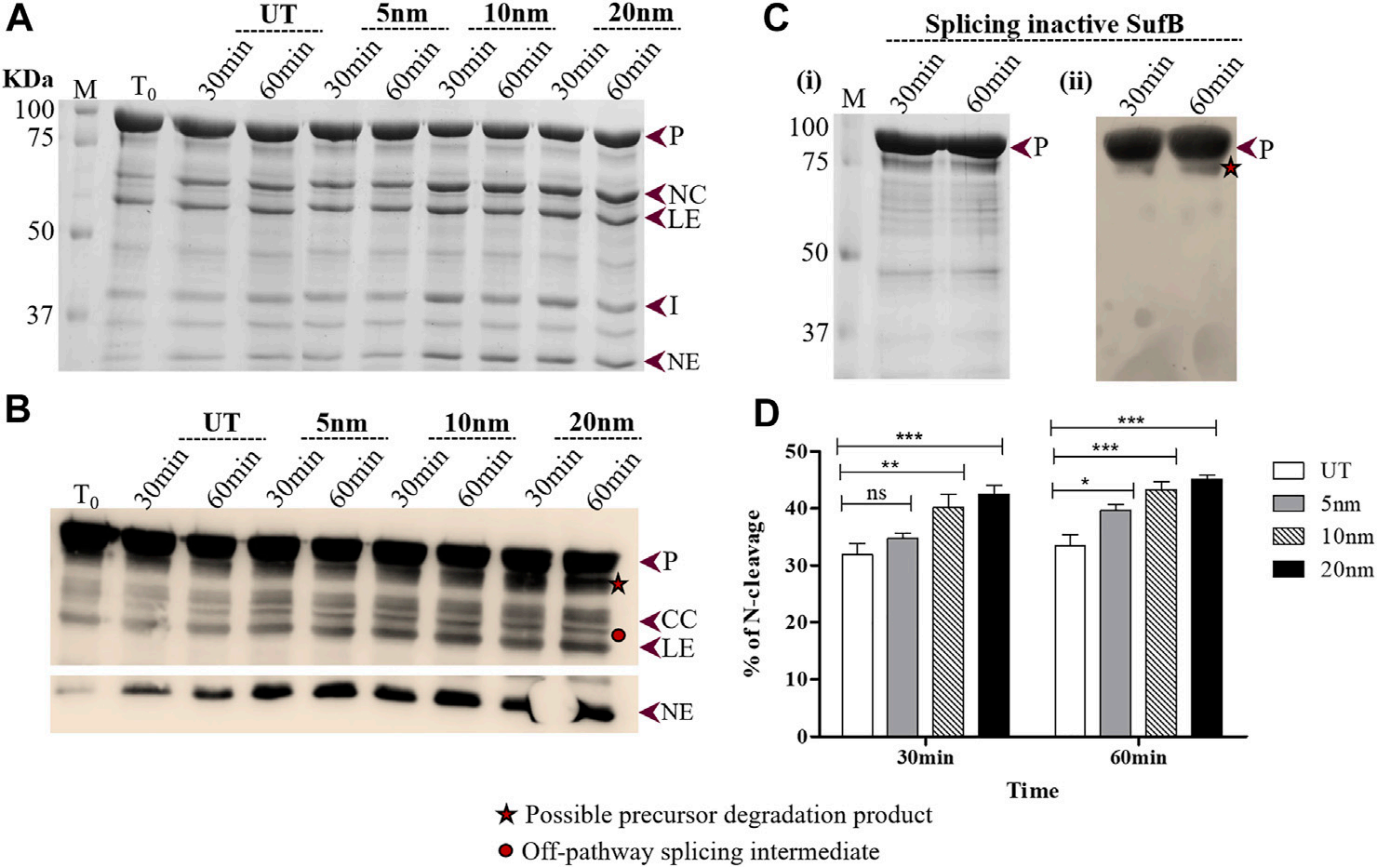
Effect of AuNP on the N-terminal cleavage reaction until 60 minutes following SufB renaturation **(A)** *In vitro* refolding of *Mtu* SufB was conducted in presence of AuNPs for a duration of 30 and 60 minutes. Resultant cleavage products were resolved through 4–10% gradient SDS-PAGE. Lane 1 (T_0_): cleavage products at time 0; Lanes 2,3 (UT): cleavage products in untreated SufB sample over specified time; Lanes 4–9: N-terminal cleavage products in presence of 0.5 ppm of AuNPs (5, 10, and 20 nm) after 30 and 60 minutes of refolding reaction. **(B)** Western blot confirms the identity of protein products from Figure 3A. Anti-His antibody detected 6x(His)-tagged P, CC, LE, and NE. NE is blotted separately with a higher concentration of primary antibody. Two additional bands were identified: one below the precursor protein (possible precursor degradation product) and the other above ligated exteins (possible off-pathway splicing intermediate). **(C)** (i) and (ii) SDS gel and western blot (anti-His antibody) images of splicing inactive SufB double mutant (C1A/N359A) that is used as a negative control. No splicing or cleavage products were observed after 30 and 60 minutes of refolding assay. (ii) **(D)** Bar graphs displaying comparative analysis of N-terminal cleavage efficiency, 30 and 60 minutes post-renaturation of SufB. There is a statistically significant increase in N-terminal cleavage reaction following treatment with AuNPs of different sizes. All the experiments were performed in triplicates and error bars represent (±1) SEM from 3 independent sets of experiments. The data shown are extracted from Figure 3A. P= Precursor, CC = C-cleavage, NC = N-cleavage, LE = ligated extein, I = intein, and NE = N-extein.

As shown earlier, binding of the proteins on the nanoparticle surface leads to conformational changes that affect protein function (Saptarshi et al., 2013; Lynch and Dawson, 2020; Park, 2020). Moreover, several parameters including size distribution, morphology, surface charge, and surface chemistry determine the biological activity of the NPs. Besides, the larger the nanoparticle, the more surface area is accessible for protein adsorption, and vice versa, resulting in efficient nanoparticle–protein interaction (Lynch and Dawson, 2008; Fei and Perrett, 2009; Woods et al., 2016; Tian et al., 2017).

In our study, AuNPs at 0.5 ppm concentration displayed an overall superior splicing efficiency than 0.25 ppm AuNPs. In the presence of 20 nm AuNPs, *Mtu* SufB exhibited 52.7% increase (*p* < 0.0001) in splicing efficiency. And 10 nm AuNPs induced 41.8% increase (*p* < 0.0001) in splicing, and 5 nm AuNPs had the least effect (24.6% increase, *p* = 0.004) on SufB splicing (Figure 1Biii). The observed size effect of AuNPs on SufB splicing is beyond the scope of our current study. However, the superior effect of the 20-nm AuNPs in the splicing reaction may be explained as follows. An efficient adsorption of SufB on the NP surface might have affected the active site residues, positively leading to splicing enhancement.

### Gold Nanoparticles Enhance N-Terminal Cleavage Reaction in *Mtu* SufB Protein

The N-terminal cleavage reaction is the first step of protein splicing which sometimes yields NC and NE as splicing by-products (Figure 1Aii). It is initiated with an N-S acyl shift by the first Cys residue (C1) of the intein, which converts the N-terminal extein–intein peptide bond to a (thio) ester linkage. As soon as transesterification via Cys+1 attack and resolution of branched chain intermediate occurs via Asn cyclization, splicing ensues, giving rise to LE and I (Figure 1Ai). Previous literature studies have described the affinity of AuNPs for cysteine thiols (Kreibig and Genzel, 1985; Aryal et al., 2006; Li et al., 2006; Petean et al., 2008; Li and Li, 2009; Ashjari et al., 2015); thus, it is likely that AuNPs interact with catalytic Cys1 to regulate N-terminal cleavage reaction, the first critical step of splicing. Further inferences were drawn by performing *in vitro* refolding and quantitative N-terminal cleavage analysis of *Mtu* SufB in the presence of AuNPs, over two different time intervals: 10–20 minutes and 30–60 minutes (Materials and Methods Section). Resultant cleavage products were resolved through 4–10% gradient SDS-PAGE and confirmed by Western blot (Figures 2, 3).

Wild-type SufB precursor gives rise to optimum N-cleavage reaction products until 60 mins after which splicing ensues (Supplementary Figure S3). Hence, the N-terminal cleavage efficiency was monitored over 10–60 mins (Figures 2, 3). After 10 mins of the refolding reaction, 20-, 10-, and 5-nm AuNP-treated *Mtu* SufB protein exhibited 42.1% (*p* < 0.001), 35.1% (*p* < 0.01), and 26.8% (*p* < 0.05) rise in the N-terminal cleavage reaction, respectively (Figure 2D). Similarly, the enhanced N-terminal cleavage reaction was noticed after 20 mins of *in vitro* SufB renaturation; 20 nm (48.8% rise, *p* < 0.001, two-way ANOVA), 10 nm (40.4% rise, *p* < 0.001, two-way ANOVA), and 5 nm (33.6% rise, *p* < 0.01, two-way ANOVA) (Figure 2D).

After 30 mins of *in vitro* refolding assay, it was observed that 20-nm AuNP-treated *Mtu* SufB had a 33.6% increase in the N-cleavage product as compared to untreated protein, which was further analyzed by two-way ANOVA (*p* < 0.0001) (Figure 3D). The 10-nm AuNP-treated SufB protein resulted in 26.4% enhancement of the N-cleavage product (*p* < 0.01, two-way ANOVA). We observed the least effect by 5-nm AuNPs (*p* < 0.05, two-way ANOVA) on the N-cleavage reaction of *Mtu* SufB protein after 30 mins of reaction. The 20- and 10-nm AuNPs maintained a significant enhancement on the N-cleavage reaction even after 60 mins of the renaturation reaction, where it resulted in 38.8% (*p* < 0.001, two-way ANOVA), and 33.2% (*p* < 0.001, two-way ANOVA) increase in the N-cleavage reaction, respectively. And 5-nm AuNP had the least effect on the N-cleavage reaction (22.1%, *p* < 0.05, two-way ANOVA) (Figure 3D). From the quantitative data, it is noticeable that the AuNPs of larger size display a consistently greater fold change in N-terminal cleavage reaction throughout 1 h. Similar experimental optimizations were maintained for all the *in vitro* refolding experiments. We hypothesize that the likely interaction of AuNPs with catalytic cysteine thiols conceivably led to the enhanced N-terminal cleavage efficiency. Thiophilic AuNPs perhaps achieve this via interaction with catalytic Cys1, which activates N-S acyl shift. Further mechanistic insights into the process are achieved via QM/MM analysis as explained later in this article.

### UV-Vis Analysis Suggests SufB intein–AuNP Interaction

SufB intein and AuNP interaction was further confirmed using UV-vis absorption spectroscopy in the wavelength ranging from 200 to 800 nm, as elaborated in the Materials and Methods section. As *Mtu* SufB precursor splices under native conditions, we have used *Mtu* SufB intein for specificity and accuracy in data analysis. SufB intein was incubated along with AuNPs (5, 10, and 20 nm) at 0.5 ppm concentration for 4 hours, and the changes in the absorption spectra were monitored.

**FIGURE 4:**
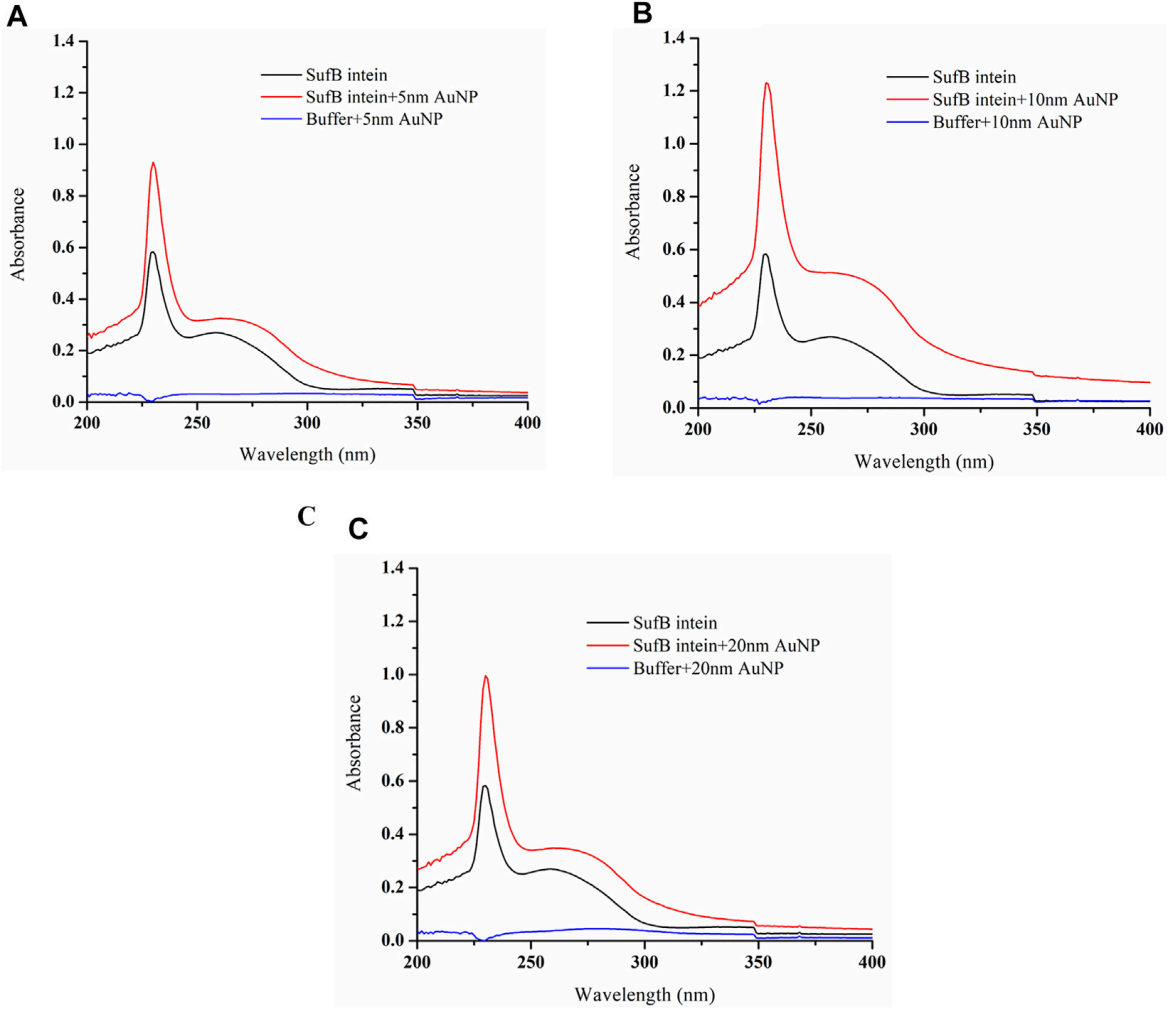
Characterization of AuNP and SufB intein interaction by UV–visible analysis. Following interaction with nanoparticles of different sizes, **(A)** 5-nm AuNP, **(B)** 10-nm AuNP, and **(C)** 20-nm AuNP samples were scanned under 200–800 nm of wavelength at constant temperature (25°C).

The untreated *Mtu* SufB intein gave an absorption peak at 258 nm. For 5- and 20-nm AuNP treated samples, we observed a minor broadening of the absorption spectra and a redshift of 2 nm (260 nm) (Figures 4A,C). As shown in Figure 4B, for the 10-nm protein–AuNP complex, we observed a distinct broadening of absorption spectra in combination with enhanced absorption, and a significant bathochromic shift. Adsorption of proteins onto AuNPs cause the peak shift due to change in the optical properties of the sample medium. The size, aggregation state, and local dielectric environment together contribute toward broadening of absorption spectra for any NP–protein complex (Suhartono et al., 2018; Randika Perera et al., 2019). In case of 10-, 20-, and 5-nm AuNPs, we see a size-dependent broadening of the peak, suggesting that protein–AuNP complex formation is optimal the 10 nm and weaker in the latter two. Our observation for peak broadening and red shift confirms the interaction between *Mtu* SufB and AuNPs for all the sizes, supported by previous observations (Giri et al., 2014). The detailed mechanisms influencing minor peak broadening by the 5- and 20-nm AuNP–protein complexes are beyond the scope of this study. Next, for a quantitative determination of binding affinity and the thermodynamic parameters of AuNP–intein interaction, we performed ITC analysis.

### ITC Determines the Binding Affinity of AuNP–*Mtu* SufB Intein Interaction

To acquire insights into the binding thermodynamics of AuNPs with SufB intein, ITC was performed. Since *Mtu* SufB precursor protein gives rise to splicing and cleavage products under native conditions, ITC was conducted with purified *Mtu* SufB intein for precision in data analysis. The measurement of ITC relies on the fact that such interactions will be accompanied by a change in heat energy. From a single titration experiment, we can obtain information about the enthalpy of binding (ΔH) and the binding constant or the affinity (K_a_). Using the equation ΔG = ΔH-TΔS, we calculated Gibb’s free energy (ΔG) and change in entropy (ΔS). The binding affinity (K_a_) is calculated from the inverse of the slope of the binding curve (Supplementary Figure S4). The shape of the binding curve also depends on the unitless parameter *c*. A high *c* value indicates tight binding, and a low *c* value depicts low-affinity interaction (Lopez and Makhatadze, 2002). The *c* value was calculated using the formula *c* = K_a_ × [P] × n, where K_a_ is the binding constant, [P] is the total protein concentration, and n is the stoichiometry of interaction (n is obtained from software), to compare the binding affinity of AuNP of different sizes (5, 10, and 20 nm) with SufB intein.

A constant volume of AuNPs was titrated against the protein at a regular interval as highlighted in the Materials and Methods section. The reaction parameters for NP–protein interactions are shown in Supplementary Figure S4. It was observed that 10-nm AuNPs have the highest binding affinity (K_a_ = 2.3 × 10^4^ M^−1^) toward SufB intein, followed by 5-nm AuNPs (K_a_ = 2.2 × 10^3^ M^−1^) and 20-nm AuNPs (Ka = 2x10^3^ M^−1^). The reaction parameters in terms of change in free energy (ΔG) were the most favorable for the 10-nm AuNPs (−5.97l kcal/mole), followed by 5-nm (−5.67l kcal/mole) and 20-nm (−5.2 kcal/mol) AuNPs (Table 1). The ΔG values, as well as the K_a_ values, show that overall AuNPs of all three sizes bind efficiently to the SufB intein system, additionally confirmed by the analysis of parameter *c*. Moreover, the binding constant values are comparable to the binding affinities of metals across various intein systems from previous studies of the literature (Table 2) (Zhang et al., 2009; Zhang et al., 2010b; Green et al., 2019). Although metals like Zn and Cu have an overall inhibitory effect upon interacting with inteins, the AuNP–*Mtu* SufB interaction is stimulatory for N-terminal cleavage and splicing reactions likely due to the thiophilic nature of AuNPs resulting in Cys1 activation. A thorough explanation of the said reaction is described in the forthcoming section (QM/MM analysis). Based on earlier research work, we can further predict that the AuNP–SufB intein binding interaction may be entropically driven owing to the endothermic nature of reaction (thermogram from Supplementary Figure S4), supported by a positive change in enthalpy (ΔH) as well as entropy (ΔS) (Supplementary Table S1) (Gorman and Amster, 1993; Zhang et al., 2009). Next, TEM analysis provided a visual confirmation for SufB intein adsorption on the AuNP surface.

**TABLE 1:** Comparative analysis of different reaction parameters obtained from the fits of ITC measurements of *Mtu* SufB intein–AuNP interaction.

	5-nm AuNP	10-nm AuNP	20-nm AuNP
Binding affinity/constant, K_a_ (M^−1^)	2.2 × 10^3^	2.3 × 10^4^	2 × 10^3^
ΔG (kcal/mol)	−5.67l	−5.97l	−5.2
ΔH (kcal/mol)	79 ± 125	80.0 ± 328	76 ± 410
c (unitless parameter)	87,120	88,8950	75,300

Reaction parameters were calculated by using the formula ΔG = ΔH-TΔS. The titration of AuNP against the protein was performed at 25°C. c is a unitless parameter that affects the shape of the binding curve. High c values indicate very tight binding, and vice versa. c = K_a_*[P_t_]*n, where K_a_ is the binding affinity, [P_t_] is the total protein concentration, and n is the stoichiometry of interaction.

**TABLE 2:** Binding affinity of Zn^2+^ and Cu^2+^ in different intein-bearing systems were obtained by using ITC and competitive metal capture analysis (CMCA). In ITC, binding affinity (K_a_) was calculated using the formula K_a_ = 1/K_d_, where K_d_ is the dissociation constant.

Material	Intein system	Dissociation constant (K_d_)	Binding affinity (K_a_)	Technique used	Reference
AuNP	*Mtu* SufB intein	5-nm AuNP- 0.45 mM	5 nm AuNP- 2.2 mM^−1^	ITC	
		10-nm AuNP- 0.042 mM	10 nm AuNP- 23.8 mM^−1^		
		20-nm AuNP- 0.48 mM	20 nm AuNP- 2.08 mM^−1^		
Zn^2+^	RecA mutants			ITC	Zhang et al. (2009)
	ΔI-SM	0.29 nM	3.4 nM^−1^		
	ΔI_hh_-SM	3.1 nM	0.3 nM^−1^		
	ΔI_hh_-CM	6.8 nM	0.14 nM^−1^		
	*Cne* Prp8 intein	1 nM	1 nM^−1^	ITC	Green et al. (2019)
	*Mtu* RecA intein	—	0.29–6.8 nM	CMCA	Zhang et al. (2010b)
Cu^2+^	*Mtu* RecA intein	—	0.098–0.16 nM	CMCA	Zhang et al. (2010b)

### TEM Analysis Reveals AuNP–Intein Corona Formation

**FIGURE 5:**
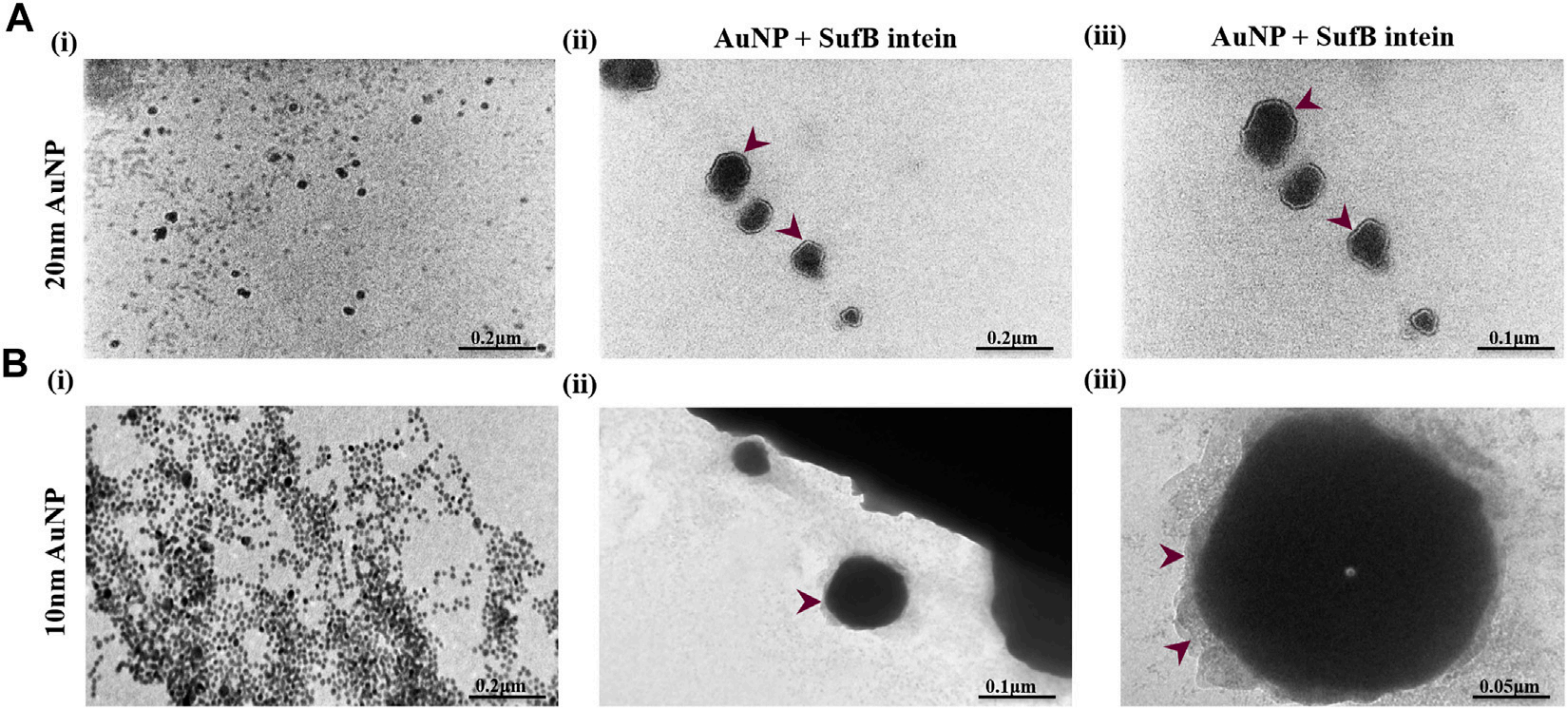
TEM images showing the formation of protein corona on **(A)** 20 nm and **(B)** 10 nm AuNP after SufB intein interaction. (i) Bare AuNP, (ii), and (iii) protein corona formation on the surface AuNPs after 4 hours of protein–AuNP incubation.

After analyzing the affinity of SufB intein towards the gold nanoparticles, we confirmed the protein–nanoparticle interaction and intein adsorption on the nanoparticle surface by TEM analysis (Figure 5). Nanoparticles of 20 and 10 nm were considered for TEM as they displayed a significant increase in splicing and N-terminal cleavage reaction of SufB protein compared to 5-nm AuNP. After protein purification, SufB intein was allowed to interact with each of the nanoparticles, as mentioned in Materials and Methods.

In Figure 5Ai, spherical and uniformly distributed 20-nm AuNPs were observed. Approximately 2-nm protein shells were formed on the surface of 20-nm AuNP (Figures 5Aii,iii), which is absent in the case of untreated AuNPs. Likewise, protein adsorption and corona formation were noticed for 10-nm AuNPs (Figures 5Bii,iii). These results support our ITC results, revealing a tightly bound layer of SufB protein on the AuNP surface even after several washing steps. Next, we performed QM/MM analysis to gather mechanistic evidence on the AuNP effect on *Mtu* SufB specifically in the N-terminal cleavage site.

### Quantum Mechanics/Molecular Mechanics (QM/MM) Analysis

The intein active site formation is crucial for the initiation of the splicing reaction (Du et al., 2009; Eryilmaz et al., 2014; Mills et al., 2014; Friedel et al., 2019b). Intein folds specifically to bring together the two splice junctions and other assistive residues to form the active site. The two splice junctions (Figure 6) are, namely, N-terminal splice junction (site) (includes Gly-1 from N-extein and Cys1 from intein) and C-terminal splice junction (site) [includes terminal Asn from intein and Cys+1 from C-extein]. The first step of the splicing reaction involves the N-terminal splice site where the N-extein is linked to the intein by a thioester bond by the critical action of Cys1 (Nanda et al., 2020). Since our experimental results have shown AuNP-induced enhancement in the N-terminal cleavage reaction, the Gly-Cys system was chosen to calculate the energy barrier using QM/MM analysis, as described in Materials and Methods.

**FIGURE 7:**
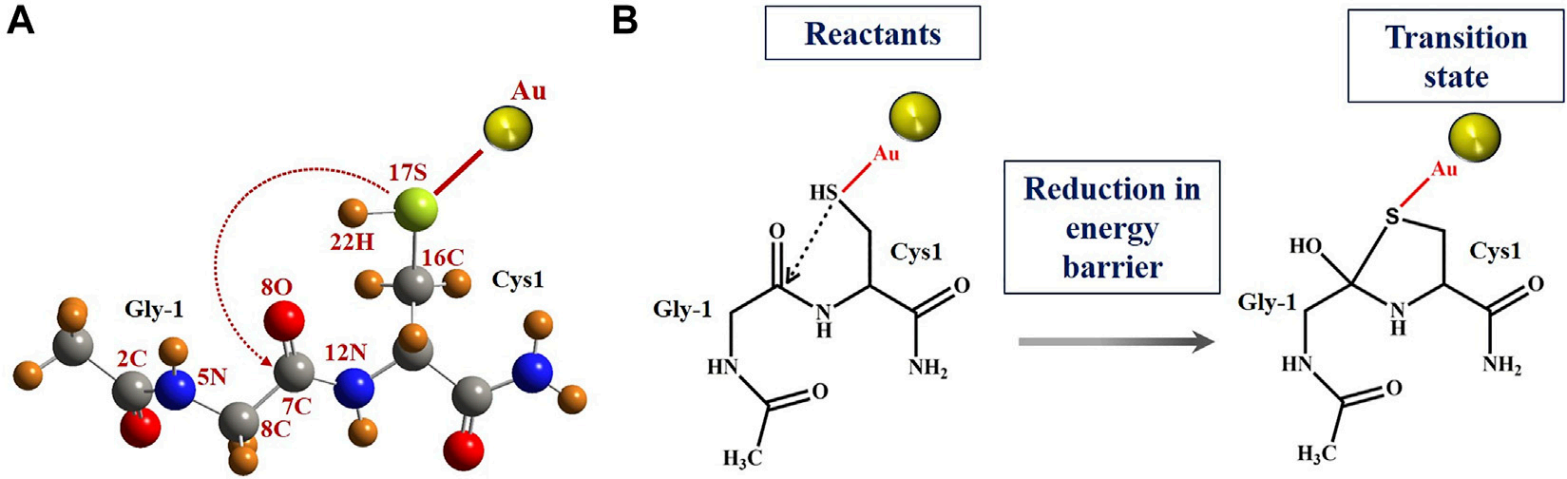
N-terminal splice site residue, that is, Gly-Cys system and N-S acyl shift reaction scheme. **(A)** Schematic geometrical structure of Gly-Cys system with Au atom as a reactant. The removal of Au provides the structure of Gly-Cys in pristine form. Oxygen, nitrogen, carbon, sulfur, gold, and hydrogen are represented with red, blue, gray, green, yellow, and orange color, respectively. The numbering of few atoms has been given for the charge analysis. **(B)** Schematic pictures of reactants and tetrahedral intermediate, respectively, of Gly-Cys system with Au atom. The reaction mechanism for N-S acyl shift between Gly-1 and Cys1 has been used based on previous report (Dearden et al., 2013). QM/MM results suggest reduction in energy barrier between the reactants and TS for the Gly-Cys system.

The schematic geometrical structure of the reactant and scheme diagram of reactants and tetrahedral intermediates is shown in Figure 7. The optimized geometrical coordinates of the structures as reactants and the transition state (TS) are given in the Supplementary Material (Pages S8-S12). During optimization, the dihedral angles of Gly-1, that is, 2C-5N-6C-7C and 5N-6C-7C-12N, were fixed at the values −121.4 and 156.5, respectively, as the surrounding protein is removed. The numbering for atoms is shown in Figure 7. Each frequency mode of the Gly-Cys system in TS has been given in Supplementary Table S2, where the presence of an imaginary mode represents that the system is in TS. The Gly-Cys system with Au is optimized (Pati et al., 2003), and TS was obtained with the aforementioned constraints and method (Materials and Methods section).

The energy difference of the reactants and TS provides the energy barrier, which is depicted in Table 3. In the vacuum, the energy barrier is found to be 0.26 eV in the presence of Au atom near by to the sulfur (of cysteine), which is comparatively much lower than the energy barrier value of 1.24 eV in the absence of the Au atom. For the realistic approach, we have performed the calculations in the solvent as well. We have used the vacuum-optimized structure of the system in the case of solvent because the calculations are computationally too expensive. However, a previous study has found that the difference in the energy between the vacuum optimized geometry and solvent optimized geometry is within the computational accuracy of 0.10 eV (Dearden, 2014). In our calculation (Table 3), the energy barrier in the case of solvent is quite different than that in the vacuum. With the inclusion of the solvent, the energy barrier is reduced from 1.24 to 0.85 eV in the absence of the Au atom. While in the case of Au atom–induced Gly-Cys system, the energy barrier is reduced from 0.26 to 0.10 eV. The reduction in the energy barrier due to solvent is associated with electrostatic screening as the interaction energy between the atoms is greatly reduced in the solvent. More importantly, in both the cases, that is, vacuum and solvent, we found that the induced Au atom reduces the energy barrier up to 0.98 eV (in vacuum) and 0.75 eV (in solvent), which possibly leads to an enhanced N-terminal cleavage reaction.

**TABLE 3:** Numerical value of the energy barrier of N-terminal splice site residues, that is, Gly-Cys (with and without Au atom) in the presence of solvent and in vacuum.

System	Energy barrier (eV) vacuum	Solvent
−Au	1.24	0.85
+Au	0.26	0.10

To elucidate the cause behind reduction in the energy barrier by Au, we investigated the electronic charge on the S (of catalytic cysteine-1), O (of carbonyl group of glycine-1), Au atoms as well as their respective nearest neighbor atoms. The Mulliken charge of the respective atoms is listed in Table 4. In this table, we have labeled the atom on the basis of our selected system as shown in Figure 7A. Table 4 shows that due to the presence of Au, the charge on other atoms, except S, is equivalent, for example, the charge on O is −0.44 (without Au) and −0.42 (with Au). There is a great decrement in the negative charge (from −0.62 to −0.28) on S, due to Au, and further, the electronic charge on the Au atom is highly negative (i.e., −0.52). The primary reason behind such changes is the lack of the π-back bonding phenomenon (Koch et al., 2019). In the π-back bonding, the electrons are channeled from the π-orbital of the ligands to the metal, in order to facilitate the bonding between ligands and the transition metal. Then the electrons from the d orbital of the transition metal are given back to the empty π* orbital of the ligand. In this process, generally, the metal atom reduces the negative charge, while the ligands enhance their negative charge. In the case of the Au atom, the d-orbital is fully filled; therefore, it does not participate in the π-back bonding process. Thus, due to the lack of π-back bonding, the Au atom contains more negative charge (−0.52), while the sulfur atom reduces the negative charge from −0.62 to −0.28. The decrement in the negative charge of the sulfur atom leads to the reduction in the repulsion between S and O atoms. The enhanced N-terminal cleavage and splicing reactions are likely due to the thiophilic nature of AuNPs that led to Cys1 activation and reduction of energy barrier in the N-cleavage site.

**TABLE 4:** Mulliken atomic charges on the atoms, that is, sulfur of catalytic cysteine and associated carbon, carbonyl oxygen of glycine and associated carbon, hydrogen, and Au atom in solvent.

Atom	Mulliken charge -Au	+Au
7C	0.62	−0.57
8O	−0.44	−0.42
16C	−0.28	−0.33
17S	−0.62	−0.28
22H	0.29	0.33
28Au		−0.52

## Conclusion

This is the first study to investigate the effect of NPs on protein splicing. We present an efficient strategy for augmenting N-terminal cleavage and splicing reactions using AuNPs of varied sizes. Time-dependent refolding studies on *Mtu* SufB protein in the presence of 20 nm AuNPs showed an overall enhanced effect on N-terminal cleavage efficiency until 1 h and splicing efficiency after 4 h of the refolding reaction. Under similar experimental conditions, 10-nm AuNPs performed better than 5-nm AuNPs. Thus, we observe a size-dependent activity of AuNPs on *Mtu* SufB splicing and cleavage reactions. UV-vis spectroscopy and ITC qualitatively and quantitatively determined the interaction between AuNPs and *Mtu* SufB protein. AuNP–protein interaction was further visualized by TEM analysis that confirmed protein corona formation. In agreement with the thiophilic nature of the gold nanoparticles, we have found a reduction in the energy barrier for the Gly-Cys system at the N-terminal splice junction that perhaps increased the N-terminal cleavage reaction and the overall splicing efficiency. Occurrence of heightened N-terminal cleavage reaction in *Mtu* SufB, as early as 10 min and conservation until 1 h under the influence of 20-nm gold nanoparticles can make it a valuable tool in the development of a novel protein purification strategy. Commercial IMPACT purification systems may take over 12 h to complete protein purification, and the DTT-mediated initiation of N- or C-terminal cleavage often runs the risk of disrupting disulfide bridges and denaturing the target protein. Undoubtedly, there is a need for extensive optimization and experimental studies to fabricate a gold nanoparticle–based purification platform. Our work also encourages thorough studies on the effect of various nanoparticles on other intein systems for the successful incorporation of nanotechnology in the fields of biomedicine, drug delivery, environmental monitoring, and imaging.

## Data Availability

The original contributions presented in the study are included in the article/Supplementary Material; further inquiries can be directed to the corresponding author.

## Abbreviations

AuNPs, gold nanoparticles; BSA, bovine serum albumin; ECL, enhanced chemiluminescence; ITC, isothermal titration calorimetry; IPTG, isopropyl β-d-1-thiogalactopyranoside; MALDI-TOF/TOF, matrix-assisted laser desorption ionization time-of-flight; *Mtu*, *Mycobacterium tuberculosis*; NPs, nanoparticles; NP-PC, nanoparticle–protein corona; PAGE, polyacrylamide gel electrophoresis; ppm, parts per million; PVDF, polyvinylidene fluoride; QM/MM, quantum mechanics/molecular mechanics; SDS, sodium dodecyl sulfate; TCEP, tris(2-carboxyethyl) phosphine; TBST, Tris-buffered saline, 0.1% Tween 20; TEM, transmission electron microscopy; UV-Vis, UV–visible spectroscopy.
