# Supplementary material for: Gold Nanoparticles Augment N-Terminal Cleavage and Splicing Reactions in Mycobacterium tuberculosis SufB
Source: Front Bioeng Biotechnol. 2021 Dec 23;9:773303. doi: 10.3389/fbioe.2021.773303 (PMC8735848; doi:10.3389/fbioe.2021.773303)
Supplement: Supplementary file 1 [file DataSheet1.pdf]

# Supplementary Material

## Gold nanoparticles augment N-terminal cleavage and splicing reactions in *Mycobacterium tuberculosis* SufB

Ananya Nanda[1], Sourya Subhra Nasker[1], Anoop K. Kushwaha[2], Deepak Kumar Ojha[1], Albert K. Dearden[3], Saroj K. Nayak[2], and Sasmita Nayak [1*]

[1]School of Biotechnology, Kalinga Institute of Industrial Technology, Bhubaneswar, Odisha, India 751024

[2]School of Basic Sciences, Indian Institute of Technology Bhubaneswar, Argul, Khurda, Odisha, India 752050

[3]Departments of Physics and Astronomy, College of Arts and Sciences, University of South Carolina, Columbia, SC 29208, United States.

*Corresponding Author: Sasmita Nayak, Email Id: sasmita.n@kiitbiotech.ac.in, Contact Number- +(91) 8763000005.

**Keywords:** Gold nanoparticles; splicing regulation; splicing enhancement; NP~intein corona; SufB; *Mycobacterium tuberculosis*

**Table of Content** **Page No.**

Gradient assay for 20nm AuNP at different concentrations S3

**Figure S1**. Gradient assay to determine the optimum concentration of AuNPs influencing splicing and cleavage reactions of *Mtu* SufB. S3

Effect of AuNPs on protein stability S4

**Figure S2**. Effect of AuNPs on the stability of precursor protein in splicing inactive SufB double mutant (SI, C1A/N359A) S4

AuNPs effect on N-terminal cleavage reaction of *Mtu* SufB until 4 hours S5

**Figure S3.** AuNPs effect on N-terminal cleavage reaction of *Mtu* SufB until 4 hours S5

Isothermal Titration Calorimetry S6

**Figure S4.** ITC titration data describing the interaction between purified SufB intein and AuNPs as titrants S6

**Table S1.** ITC data depicts a positive change in entropy (ΔS) during AuNP-SufB intein interaction S7

Identification of splicing and cleavage products of *Mycobacterium tuberculosis* full-length SufB protein using MALDI-TOF Mass spectrometry S7

Geometrical coordinates of *Mtu* SufB optimized structure S8-S12

**Table S2.** Frequency for transition state S13- S22

## Gradient assay for 20nm AuNP at different concentrations

We performed gradient assay to determine the optimum concentration of AuNPs that can enhance the splicing and cleavage reactions in *Mtu* SufB. *In vitro* refolding of denatured SufB protein was carried out for a period of 4hrs in presence of 20nm AuNP at various concentrations ranging from 0.05ppm to 2ppm. The details of the experimental steps are mentioned in the main text (Methods section). 0.25ppm and 0.5ppm concentrations were selected for the remaining *in vitro* studies.

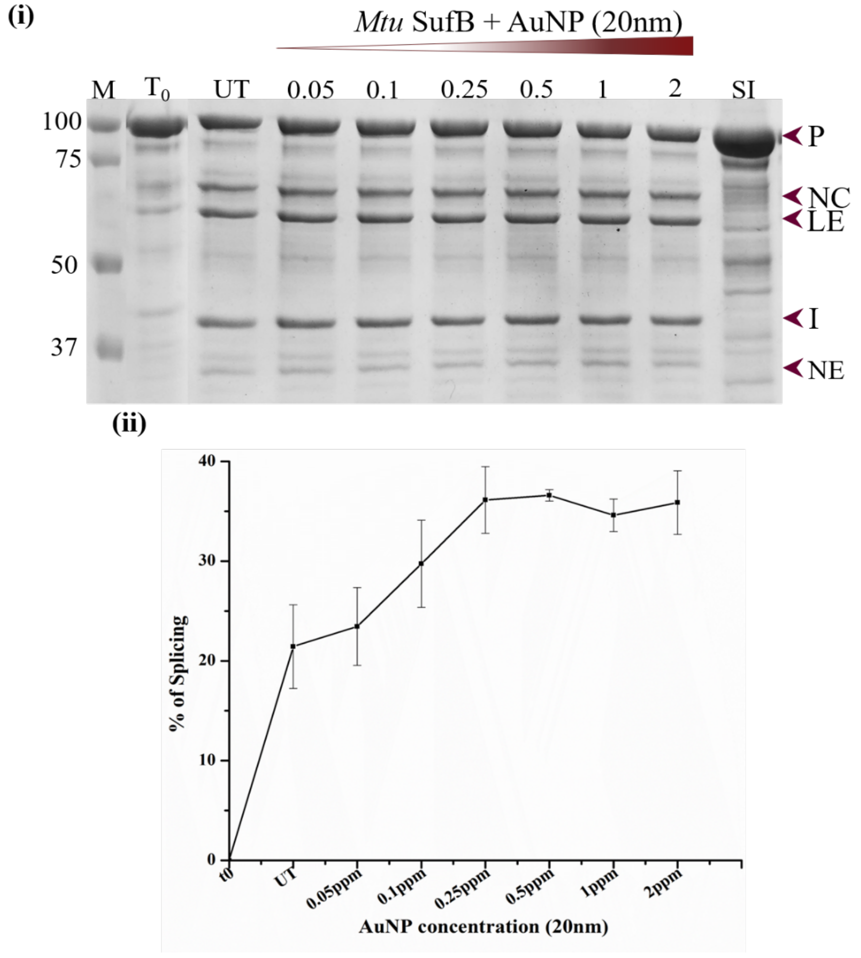


**Figure S1. (i) Gradient assay to determine the optimum concentration of AuNPs influencing splicing and cleavage reactions of *Mtu* SufB.** Following *in vitro* refolding of *Mtu* SufB precursor protein in presence of 20nm AuNP at varied concentrations (0.05ppm-2ppm), resultant products were resolved through 4~10% gradient SDS-PAGE. Lane 1 ($T_{0}$): splicing products at time 0; Lane 2 (UT): splicing products in untreated SufB sample; Lanes 3-8: splicing products resulting in presence of AuNPs (20nm) at different concentration (0.05ppm to 2.0 ppm). Lane 9 (SI): splicing inactive SufB double mutant (C1A/N359A) is used as a negative control for splicing. **(ii)** Line plot representing

splicing efficiency of *Mtu* SufB in presence of AuNPs at different concentrations relative to untreated (UT) protein. The experiments were performed in triplicates and error bars represent (±1) SEM from 3 independent sets of experiments. The data shown are extracted from Figure S1(i). P= Precursor, CC= C-cleavage, NC= N-cleavage, LE= Ligated extein, I= intein, NE= N-extein.

**Effect of AuNPs on protein stability**

To check the effect of AuNPs on the stability of test protein, we performed *in-vitro* splicing analysis of splicing inactive SufB double mutant (SI, C1A/N359A) in presence of AuNPs of different sizes (5nm, 10nm, 20nm) over a period of 4hrs at 25$^0$C. The details of experimental procedures are described in the main text (Methods section). Substitution of catalytic residues Cys1 and Asn359 to alanine resulted in complete inhibition of intein splicing as reported by earlier works. Un-spliced SufB precursor protein was visualized via SDS-PAGE and further confirmed by western blot using anti-His antibody. We did not observe any protein degradation in presence of AuNPs during the specified time.

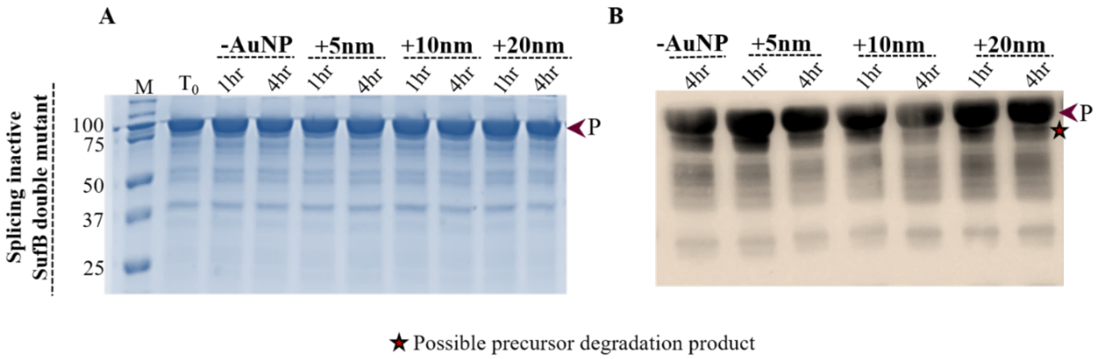


**Figure S2. Effect of AuNPs on the stability of precursor protein in splicing inactive SufB double mutant.** (A) SDS-PAGE image displaying no effect of AuNPs on splicing inactive SufB double mutant (SI, C1A/N359A). (B) Anti-His antibody detects 6X (His)-tagged SufB precursor protein via western blot.

**AuNPs effect on N-terminal cleavage reaction of *Mtu* SufB until 4 hours**

Effect of AuNPs on the first amide-thioester reaction of *Mtu* SufB was assessed over a period of 4hrs. Details of the experimental procedures are mentioned in the main text (Methods sections). The resultant products were resolved through 4-10% gradient SDS-PAGE. Trans-esterification is the first step of SufB splicing which is initiated by Cys1 with N-S acyl shift. We could visualize N- terminal cleavage products (NC, N-cleavage product, and NE, N-extein) until 240 mints (4hours), albeit splicing dominated after 60 mints. Hence the N-terminal cleavage reaction in *Mtu* SufB was monitored until 60 mints as mentioned in the main text. Splicing inactive *Mtu* SufB double mutant and SufB intein (treated under similar experimental conditions) were used as the negative control for our study. Anti-(His) antibody detected the presence of 6x (His)-tagged P, I, and an additional band below the precursor protein (possible precursor degradation product), which was absent for SufB intein.

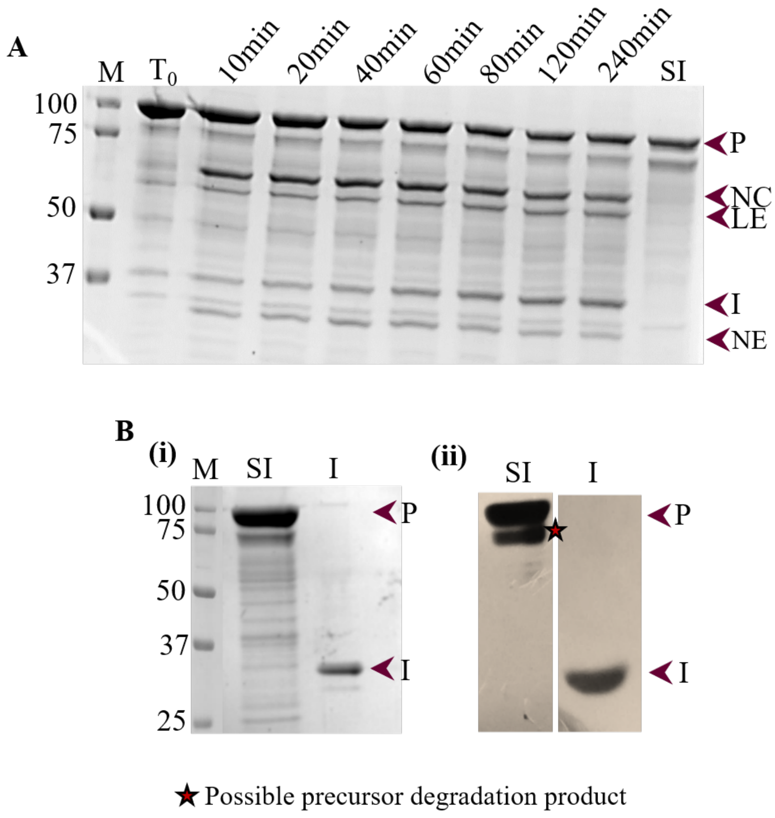


**Figure S3. AuNPs effect on N-terminal cleavage reaction of *Mtu* SufB until 4 hours. (A)** Products resulting from 4hrs (240mins) of refolding of *Mtu* SufB were resolved through 4~10% gradient SDS-PAGE. Splicing inactive SufB double mutant (SI, C1A/N359A) was used as negative control. P= Precursor, NC= N-cleavage, LE= Ligated extein, I= intein, NE= N-extein. **(B)** (i) SDS-PAGE showing the precursor protein of splicing inactive SufB double mutant (SI, C1A/N359A) and SufB intein, (ii) Western blot confirms the identity of protein products from Figure B(i). Anti-(His) antibody detected the

presence of 6x (His)-tagged P and I. An additional band was identified below the precursor protein (possible precursor degradation product), which was absent for SufB intein. P= Precursor, I= intein.

## Isothermal Titration Calorimetry (ITC)

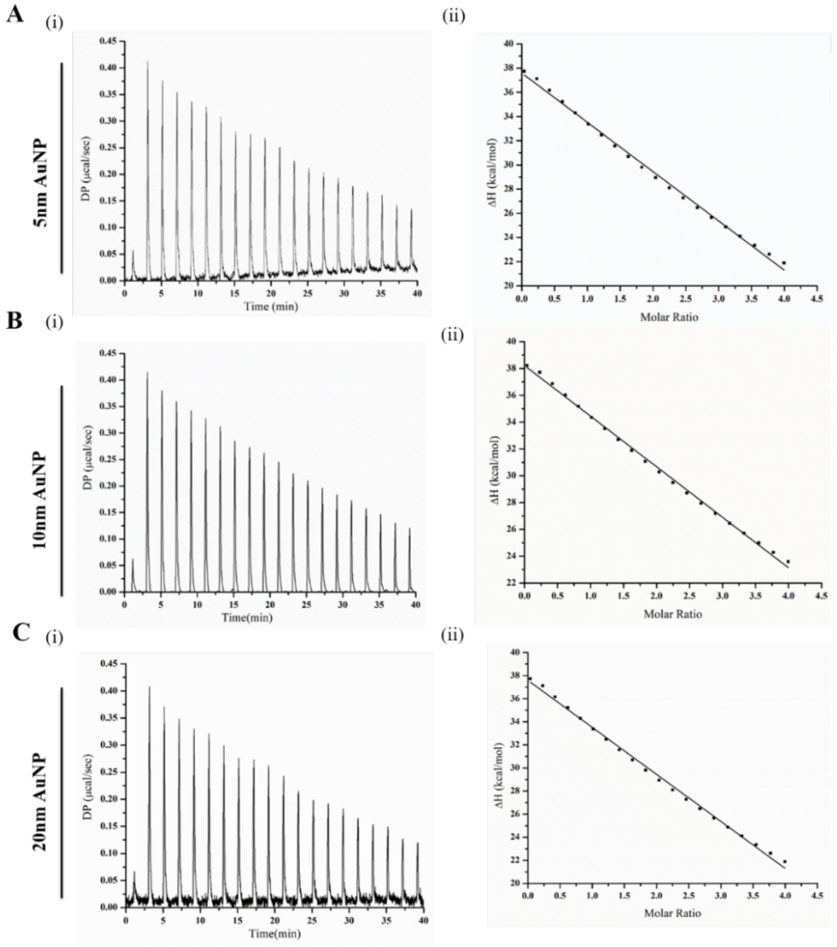


**Figure S4. ITC titration data describing the interaction between purified SufB intein and the titrants** (A) 5nm AuNP, (B) 10nm AuNP and (C) 20nm AuNP at 25˚C. The column (i) represents raw calorimetric data obtained by titration of respective nanoparticles against the protein in the sample cell and column (ii) represents the integrated normalized data of the curves shown in the left panel respectively plotted as a function of molar ratio of respective nanoparticles added to the reaction cell during titration of respective AuNPs.

The details of the ITC experimental procedures are provided in the main text (Methods section).

**Table S1. ITC data depicts a positive change in entropy (ΔS) during AuNP-SufB interaction**

ΔS is calculated using the formula ΔG = ΔH-TΔS, where T is conserved at 298.15K throughout the experiments. We found a positive change in entropy, which might indicate an entropy-driven interaction between AuNPs and SufB intein.

| | 5nm AuNPs | 10nm AuNPs | 20nm AuNPs |
|---|---|---|---|
| ΔS (kcal/mol) | 0.28 | 0.29 | 0.27 |

**Identification of splicing and cleavage products of *Mycobacterium tuberculosis* full-length SufB protein using MALDI-TOF Mass spectrometry**

MALDI-TOF mass spectrometry was performed to identify different splicing and cleavage products of *Mycobacterium tuberculosis* SufB protein. The data has been deposited to the ProteomeXchange Consortium via PRIDE partner repository with data set identifier PXD015199. The details of the submission are given below

**Project Name**: Identification of full length, splicing and cleavage products of SufB protein of SUF-complex of *Mycobacterium tuberculosis*.

**Project accession**: PXD015199

Reviewer account details:

**Username**- reviewer73867@ebi.ac.uk; **Password**: bRxRcbvP

**Geometrical coordinates of *Mtu* SufB optimized structure**

**1. Without Au atom**

**1.1 Reactant**

| Symbol | X | Y | Z |
|---|---|---|---|
| C | 5.0940370 | 1.1603700 | -0.2492440 |
| C | 4.1056150 | 0.0090590 | -0.3383570 |
| O | 4.1683830 | -0.8345280 | -1.2242250 |
| H | 4.9310790 | 1.8133190 | 0.6119550 |
| N | 3.1668990 | -0.0322470 | 0.6618440 |
| C | 2.0409580 | -0.9358950 | 0.5900300 |
| C | 0.7317730 | -0.1457040 | 0.6449340 |
| O | 0.6767990 | 0.9687070 | 1.1660880 |
| H | 3.0033150 | 0.7977220 | 1.2176500 |
| H | 2.1351320 | -1.5143110 | -0.3331250 |
| H | 2.0443260 | -1.6455180 | 1.4300610 |
| N | -0.3485230 | -0.7615110 | 0.1062850 |
| C | -1.6872150 | -0.2256140 | 0.2531600 |
| C | -2.6543290 | -1.3990380 | -0.0186340 |
| O | -2.2302170 | -2.4903360 | -0.3866060 |
| C | -1.8877370 | 0.9780560 | -0.7033090 |
| S | -3.3545370 | 2.0366820 | -0.3447360 |
| H | -0.3137860 | -1.7137370 | -0.2461920 |
| H | -1.8214630 | 0.1299200 | 1.2822940 |
| H | -0.9985720 | 1.6050900 | -0.6470840 |
| H | -2.0076940 | 0.6383020 | -1.7352610 |
| H | -2.8571370 | 2.6134420 | 0.7685350 |
| N | -3.9663200 | -1.1423420 | 0.1941900 |

| | | | |
|---|---|---|---|
| H | -4.2923260 | -0.1953930 | 0.3430970 |
| H | 6.1059280 | 0.7498990 | -0.1992900 |
| H | 5.0275310 | 1.7515830 | -1.1666800 |
| H | -4.6264710 | -1.8626770 | -0.0599530 |

### 1.2 Transition State

| Symbol | X | Y | Z |
|---|---|---|---|
| C | 4.9815740 | 0.5283980 | -0.4809800 |
| C | 3.8202980 | -0.4320320 | -0.3053940 |
| O | 3.7160470 | -1.4842660 | -0.9185800 |
| H | 4.6289870 | 1.5396220 | -0.7090100 |
| N | 2.8898340 | -0.0419000 | 0.6358770 |
| C | 1.6799520 | -0.7986210 | 0.8259570 |
| C | 0.4309870 | 0.0309890 | 0.6029600 |
| O | 0.5463320 | 1.2912830 | 0.8185460 |
| H | 2.9293060 | 0.8881380 | 1.0271260 |
| H | 1.7184030 | -1.6462200 | 0.1352730 |
| H | 1.6111230 | -1.2047550 | 1.8465570 |
| N | -0.6772520 | -0.5898660 | 0.2826270 |
| C | -2.0747430 | -0.1195820 | 0.2224340 |
| C | -2.8659390 | -1.4206230 | -0.0414810 |
| O | -2.2873900 | -2.4889680 | -0.2386280 |
| C | -2.3227770 | 0.9982080 | -0.8190880 |
| S | -1.7427650 | 2.6392890 | -0.2467820 |
| H | -0.6421830 | -1.5967350 | 0.0794870 |

| | | | |
|---|---|---|---|
| H | -2.3542030 | 0.2745890 | 1.2066360 |
| H | -1.8632620 | 0.6872220 | -1.7657340 |
| H | -3.4025500 | 1.0454930 | -1.0020710 |
| H | -0.2092920 | 1.8271180 | 0.5307710 |
| N | -4.2101340 | -1.3014150 | -0.0191640 |
| H | -4.6673680 | -0.4088670 | 0.0793000 |
| H | 5.5783450 | 0.5793230 | 0.4358330 |
| H | 5.6089960 | 0.1705830 | -1.2962810 |
| H | -4.7652230 | -2.1166840 | -0.2319070 |

## 2 With Au atom

### 2.1 Reactant

| Symbol | X | Y | Z |
|---|---|---|---|
| C | -5.7388430 | -3.0472750 | 0.0556530 |
| C | -5.4532280 | -1.6210220 | -0.3839930 |
| O | -5.7123140 | -1.2211730 | -1.5120250 |
| H | -5.5408510 | -3.2277310 | 1.1154060 |
| N | -4.9087800 | -0.8096900 | 0.5822230 |
| C | -4.3906080 | 0.4963910 | 0.2454790 |
| C | -2.9142310 | 0.5935170 | 0.6295920 |
| O | -2.4269590 | -0.1113120 | 1.5134830 |
| H | -4.5015360 | -1.2297800 | 1.4077700 |
| H | -4.5490850 | 0.6502990 | -0.8257740 |
| H | -4.9308100 | 1.2917380 | 0.7797530 |
| N | -2.1881430 | 1.5208730 | -0.0459820 |

| | | | |
|---|---|---|---|
| C | -0.8272780 | 1.8352280 | 0.3323040 |
| C | -0.4942430 | 3.1965570 | -0.3180820 |
| O | -1.2605770 | 3.7079490 | -1.1259260 |
| C | 0.1185630 | 0.6971740 | -0.1309110 |
| S | 1.7595000 | 0.6939060 | 0.7340820 |
| H | -2.5912500 | 2.1376440 | -0.7449610 |
| H | -0.7672570 | 1.9155370 | 1.4250000 |
| H | -0.3543420 | -0.2620040 | 0.0740220 |
| H | 0.3416740 | 0.7666010 | -1.1977790 |
| H | 1.3258340 | 0.1170400 | 1.8762710 |
| N | 0.6754480 | 3.7650230 | 0.0619770 |
| H | 1.3255050 | 3.3007110 | 0.6791520 |
| H | -6.7858760 | -3.2763520 | -0.1577320 |
| H | -5.1277320 | -3.7289130 | -0.5432530 |
| H | 0.9513090 | 4.6233230 | -0.3925540 |
| Au | 3.0557060 | -0.9800150 | -0.1647170 |

**2.2 Transition state**

| Symbol | X | Y | Z |
|---|---|---|---|
| C | 6.1459630 | -2.0075420 | 0.3024470 |
| C | 5.4330270 | -0.6935220 | 0.0460420 |
| O | 5.8505940 | 0.3897120 | 0.4272250 |
| H | 5.4979220 | -2.7066650 | 0.8415900 |
| N | 4.2612920 | -0.8062720 | -0.6765510 |
| C | 3.4571930 | 0.3534520 | -0.9521610 |
| C | 2.0453430 | 0.2397190 | -0.4176960 |

| | | | |
|---|---|---|---|
| O | 1.5795740 | -0.9601520 | -0.3009830 |
| H | 3.8992160 | -1.7160850 | -0.9206950 |
| H | 3.9718740 | 1.2108220 | -0.5076030 |
| H | 3.3696920 | 0.5413580 | -2.0334380 |
| N | 1.3663200 | 1.3321220 | -0.1758740 |
| C | -0.0680550 | 1.4977010 | 0.0930010 |
| C | -0.2965430 | 3.0167860 | -0.0189120 |
| O | 0.6551970 | 3.7935900 | -0.0902380 |
| C | -0.5267020 | 0.8976140 | 1.4452190 |
| S | -0.8801830 | -0.9085000 | 1.3223790 |
| H | 1.8239100 | 2.2502910 | -0.2467530 |
| H | -0.6452970 | 1.0017560 | -0.7010010 |
| H | 0.2411180 | 1.0709240 | 2.2055330 |
| H | -1.4338640 | 1.4109840 | 1.7682920 |
| H | 0.7239080 | -1.0388220 | 0.1490690 |
| N | -1.5922570 | 3.3873860 | -0.0349750 |
| H | -2.3360000 | 2.7026400 | -0.0951890 |
| H | 6.4381580 | -2.4802470 | -0.6412180 |
| H | 7.0375020 | -1.8102680 | 0.8964000 |
| H | -1.8124530 | 4.3660340 | -0.1475510 |
| Au | -2.5666400 | -0.8008840 | -0.2378400 |

**Table S2. Frequency for Transition state**

**1. In absence of Au atom**

| Frequency | IR Intensity |
|---|---|
| -331.27 | 27.954 |
| 18.03 | 3.7911 |
| 32.64 | 2.6204 |
| 62.61 | 14.7901 |
| 79.02 | 5.4091 |
| 111.34 | 12.4719 |
| 126.70 | 9.5688 |
| 170.08 | 6.3894 |
| 201.15 | 2.6586 |
| 222.32 | 14.5501 |
| 261.10 | 27.8908 |
| 264.11 | 26.1213 |
| 281.07 | 8.4155 |
| 323.26 | 230.8521 |

| | |
|---|---|
| 329.07 | 11.9367 |
| 369.04 | 15.7529 |
| 396.38 | 12.7229 |
| 451.90 | 17.9421 |
| 461.27 | 129.7181 |
| 520.06 | 11.8002 |
| 549.63 | 7.4593 |
| 602.70 | 6.8652 |
| 614.67 | 10.3434 |
| 625.25 | 17.9212 |
| 642.72 | 5.8579 |
| 690.68 | 4.9068 |
| 708.01 | 2.8528 |
| 763.85 | 5.1355 |
| 831.55 | 3.6885 |
| 851.74 | 48.7945 |

| | |
|---|---|
| 889.66 | 6.0956 |
| 913.90 | 8.1171 |
| 960.35 | 14.9606 |
| 981.92 | 16.587 |
| 999.98 | 7.2138 |
| 1041.63 | 7.0723 |
| 1057.48 | 7.991 |
| 1059.37 | 16.6266 |
| 1116.34 | 14.3827 |
| 1137.56 | 56.3001 |
| 1170.25 | 7.3934 |
| 1186.55 | 4.6194 |
| 1241.64 | 14.1737 |
| 1254.06 | 15.224 |
| 1271.32 | 204.6729 |
| 1275.51 | 51.7831 |
| 1328.95 | 18.1136 |

| | |
|---|---|
| 1353.72 | 98.5206 |
| 1390.74 | 83.0316 |
| 1407.73 | 2.8189 |
| 1436.41 | 12.8931 |
| 1475.80 | 34.4981 |
| 1481.82 | 29.8312 |
| 1486.68 | 0.566 |
| 1497.70 | 9.7275 |
| 1542.91 | 285.322 |
| 1586.04 | 230.5898 |
| 1630.38 | 157.5847 |
| 1691.49 | 310.003 |
| 1784.02 | 180.7622 |
| 1796.49 | 234.4491 |
| 3013.96 | 30.8621 |
| 3027.60 | 65.4419 |

| | |
|---|---|
| 3056.09 | 3.7725 |
| 3058.78 | 5.19 |
| 3073.11 | 26.6409 |
| 3112.60 | 24.4644 |
| 3120.74 | 8.1113 |
| 3181.02 | 4.6593 |
| 3270.28 | 2496.6367 |
| 3376.58 | 447.7503 |
| 3603.98 | 65.3576 |
| 3642.94 | 40.3679 |
| 3739.83 | 57.9906 |

**2. In presence of Au atom**

| Frequency | IR intensity |
|---|---|
| -105.74 | 1.7902 |
| 28.19 | 3.4952 |
| 46.48 | 1.6636 |

| | |
|---|---|
| 60.10 | 14.294 |
| 92.63 | 1.5731 |
| 119.14 | 4.2336 |
| 133.63 | 20.3757 |
| 179.48 | 2.2427 |
| 209.38 | 2.2437 |
| 244.69 | 10.4196 |
| 261.94 | 13.8617 |
| 277.99 | 0.1682 |
| 310.72 | 8.3301 |
| 332.50 | 12.8678 |
| 350.61 | 17.1192 |
| 388.54 | 5.9704 |
| 424.34 | 49.0509 |
| 426.56 | 248.8399 |
| 433.35 | 19.5692 |

| | |
|---|---|
| 459.23 | 46.2531 |
| 531.19 | 7.76 |
| 573.96 | 0.6351 |
| 602.91 | 8.0321 |
| 615.31 | 10.9746 |
| 629.71 | 11.6051 |
| 647.06 | 5.8176 |
| 685.25 | 3.5479 |
| 709.83 | 3.5236 |
| 758.91 | 3.427 |
| 840.88 | 2.6264 |
| 851.26 | 46.9345 |
| 895.17 | 19.3166 |
| 911.04 | 3.7648 |
| 959.48 | 23.8373 |
| 983.95 | 21.7698 |
| 1007.09 | 6.9872 |

| | |
|---|---|
| 1042.43 | 7.9808 |
| 1058.90 | 4.9492 |
| 1079.30 | 8.6907 |
| 1127.52 | 7.6616 |
| 1157.75 | 85.5599 |
| 1171.41 | 3.2893 |
| 1194.49 | 27.0588 |
| 1251.19 | 0.8041 |
| 1257.44 | 26.716 |
| 1272.88 | 305.1749 |
| 1295.94 | 6.2572 |
| 1320.51 | 65.9869 |
| 1351.64 | 63.0592 |
| 1392.23 | 78.109 |
| 1408.88 | 2.6722 |
| 1440.80 | 10.3987 |

| | |
|---|---|
| 1470.55 | 22.5603 |
| 1479.18 | 12.9001 |
| 1480.27 | 34.3017 |
| 1495.88 | 10.0292 |
| 1542.83 | 282.5699 |
| 1560.93 | 84.6096 |
| 1635.17 | 138.3112 |
| 1689.78 | 252.0304 |
| 1793.34 | 189.4556 |
| 1801.21 | 284.1832 |
| 3003.47 | 79.7473 |
| 3013.29 | 22.3061 |
| 3057.79 | 3.3251 |
| 3077.85 | 11.1406 |
| 3113.48 | 27.6475 |
| 3121.96 | 7.5636 |
| 3139.03 | 2.281 |

| | |
|---|---|
| 3183.60 | 3.9139 |
| 3355.18 | 761.3034 |
| 3386.96 | 578.7667 |
| 3555.71 | 62.0103 |
| 3652.24 | 35.1176 |
| 3705.94 | 125.9315 |
